# Myofunctional Therapy and Its Effects on Retropalatal Narrowing and Snoring: A Preliminary Analysis of Rehabilitative Approaches

**DOI:** 10.7150/ijms.113922

**Published:** 2025-07-21

**Authors:** Vitriana Biben, Fatrika Dewi, Shinta Sari Ratunanda, Nushrotul Lailiyya, Sitti Ayu Hemas Nurarifah, Nabilla Fikria Alviani

**Affiliations:** 1 Physical Medicine and Rehabilitation Department, Faculty of Medicine, Universitas Padjadjaran/dr.Hasan Sadikin General Hospital, Bandung, West Java, Indonesia.; 2 Ear, Nose, and Throat-Head and Neck Surgery Department, Faculty of Medicine, Universitas Padjadjaran/dr.Hasan Sadikin General Hospital, Bandung, West Java, Indonesia.; 3 Neurology Department, Faculty of Medicine, Universitas Padjadjaran/dr.Hasan Sadikin General Hospital, Bandung, West Java, Indonesia.

**Keywords:** Myofunctional therapy exercise, pharyngeal area collapse, snoring.

## Abstract

**Introduction:** Snoring is a common and disturbing condition that is associated with sleep apnea in some cases. Retropalatal narrowing has been reported to be often associated with the condition, as it can lead to increased air turbulence and vibration of tissues in the throat during breathing. The activation of the tongue, soft palate, lateral wall of the pharynx, and face has been shown to keep the upper muscles open during sleep, potentially improving snoring. Therefore, this study aims to determine the effects of myofunctional therapy (MT) in patients with collapsed pharynx.

**Methods:** A total of 18 participants were selected based on inclusion and exclusion criteria in this one-group pre-post study. MT exercise intervention was performed for 6 weeks, and flexible nasolaryngoscopy was used to diagnose the area of pharyngeal collapse. The impact of MT was assessed using the Berlin questionnaire (to assess the frequency and intensity of snoring) and the Epworth Sleepiness Scale (to assess daytime sleepiness), which were filled out before and after exercise.

**Results:** The results showed a significant decrease in the percentage of narrowing of pharyngeal area (retropalatal) (58.8%, p < 0.05) after the intervention, as well as the frequency of snoring (94.1%, p < 0.01). In addition, there was a decrease in snoring frequency, improvement in snoring intensity (100%, p < 0.01), and decline in daytime sleepiness (mean 3.59±2.740, p < 0.01).

**Conclusion:** MT can effectively alleviate narrowing in pharyngeal area, leading to a reduction in snoring and daytime sleepiness

## Introduction

Sleep is a crucial activity that occupies about one-third of human life, while snoring is the most frequently reported issue in sleep medicine clinics. Snoring is a type of breathing disturbance known as sleep-disordered breathing. Several studies have shown that it can lower sleep quality and is harmful to the health. The International Classification of Sleep Diseases, Third Edition (ICSD-3) defines this condition as audible vibrations of the upper airway during respiration 1,2. In addition, the condition is widespread among the general population. Previous studies have shown that women are more likely to experience snoring after the age of 65, with an increase following menopause, while it is often experienced by men between the ages of 45 and 64 1. The age group between 45 and 54 years old has the highest prevalence of snoring, but there is no available data in Indonesia 3.

The vibrating of soft tissue structures during inspiratory airflow via a constricted (narrowed) upper airway due to upper airway collapse/obstruction is the common cause of snoring 2,4. Patients can experience snoring as a symptom of a sleep disease, such as Obstructive Sleep Apnea (OSA), or as an independent phenomenon. Primary snoring in the absence of OSA diagnosis is common and may be linked to negative health risks, such as increased carotid intima media thickness, elevated levels of systemic inflammatory markers, and higher healthcare utilization. In addition, it can adversely affect the sleep quality of those sharing the bed, lead to daytime drowsiness, strain relationships, cause social embarrassment, and disrupt mental health, ultimately diminishing overall quality of life 4.

The screening questionnaire for OSA must be accessible to perform, precise, and appropriate for different populations. The majority of existing studies focused on the validation of questionnaires in sleep clinic patients, where the prevalence of OSA is high. Sleep clinics may demand questionnaires of high sensitivity, such as STOP-Bang Questionnaire (SBQ), to accurately diagnose patients with OSA. When the result of SBQ is 5 or higher, it may prompt clinicians to carry out Polysomnography (PSG). This is because the higher the score, the greater the probability of severe OSA. In some populations, such as patients undergoing surgery, time of predicting OSA is crucial. SBQ is a quick and verified tool for predicting this disease and is clinically convenient and applicable under time-sensitive situation. In the general population, the high specificity of questionnaire may prevent unnecessary referral for PSG 2,4.

Anatomical risk factors are believed to be significant contributors to upper airway constriction during sleep. Features, such as a large neck circumference, excess soft tissue, bone structure, or arterial presence can promote pharyngeal narrowing. These structures may cause the upper airway's surrounding pressure to rise, which causes pharyngeal collapsibility or a lack of room to allow for the airflow in a section during sleep. Hypotonia of the upper airway muscles is also a significant factor because a repeated whole or partial airway collapse occurs as it becomes weaker 5. Collapse can occur at many levels, including retropalatal level (soft palate), retroglossal level (base of tongue), lateral wall of the oropharynx, and hypopharynx (epiglottis) 6. Flexible nasolaryngoscopy (FN) offers an alternative to direct airway visualization, examining the airway from the nasal cavity to the larynx. It is not only easier to perform and more cost-effective but also allows for a thorough examination of the airway to more precisely pinpoint the location of pharyngeal narrowing that causes obstruction 7.

Treatment options for snoring include lifestyle changes (like reducing alcohol consumption or losing weight), positional therapy, mandibular advancement devices, upper airway surgery, and nasal continuous positive airway pressure. In addition, myofunctional therapy (MT) can serve as an adjunct or alternative treatment, potentially reducing the Apnea-Hypopnea Index in OSA patients who experience collapse of pharyngeal dilator muscles 8. This exercise is a less invasive approach that involves exercising the tongue, soft palate, and pharynx lateral wall 8. Exercise is non-invasive, cost-effective, and offers physiological benefits. Enhancements in upper airway obstruction are supported by increases in the tone of the muscles characterized by the addition of retropalatal distance and shortening of the soft palate in MRI results 9. It also proved to improve respiratory function, mastication, swallowing, speech, daytime sleepiness, and snoring over time by increasing endurance, neuromuscular coordination, stability, and pharyngeal dilator muscle tone 10. While these treatments may be helpful, the effect of MT on retropalatal narrowing, which is one of the causes of snoring, needs further clarification 1. Therefore, this study aims to assess the effect of MT on patients who snore and have collapsed pharynx, as identified by a FN examination.

## Methods

This study used a group pretest-posttest approach, and the subjects were selected from secondary medical record data through consecutive sampling. Males, 20 to 65 years old, showed a high risk of OSA SBQ ≥ 3 and Berlin Questionnaire score positive in ≥ 2 categories), already diagnosed by an ENT (Ear, Nose and Throat) specialist as having a risk of OSA, as showed by the questionnaire and FN study, which showed collapse of pharyngeal muscles in the velopharyngeal or retropalatal area since September 2022 until February 2023. The accessible population was RSHS employees with a high risk of OSA based on secondary data from medical records at Hasan Sadikin Hospital. The sampling method used was consecutive sampling, and the investigators contacted subjects who met the acceptance criteria. The study subjects who were willing to participate in the reports were directed to sign an informed consent form. These could be excluded when having a history of lung disease, craniofacial anatomical abnormalities, or when using sedative drugs, alcohol, antidepressants, or anti-anxiety medications in the 3 months before their selection. Subjects who met the inclusion and exclusion criteria were then contacted by the investigators and directed to sign informed consent when participating in the study.

This study used a diagnostic procedure with an FN accompanied by Muller's Maneuver (MM) that was performed in awake conditions, to determine the obstruction or upper airway collapsibility in all subjects. Furthermore, there were 4 levels of upper airway collapse assessed by MM, which included Muller 1 (base of tongue, anteroposterior collapse, retroglossal level), Muller 2 (pharyngeal lateral wall collapse, retroglossal level), Muller 3 (soft palate, anteroposterior collapse, retropalatal level), and Muller 4 (pharyngeal lateral wall collapse, retropalatal level). The degree of obstruction was scored semi-quantitatively as 0 = no obstruction, 1 = 25%, 2 = 50%, 3 = 75%, and 4 = 100% 11.

Duration and length of exercise referred to Ye et al.'s study**^9^** which showed significant results with exercise for 6 weeks, 2 times per day for 20 minutes. MT carried out training some muscles with some stimulation, as outlined below:

### Soft palate

**Figure 1:**
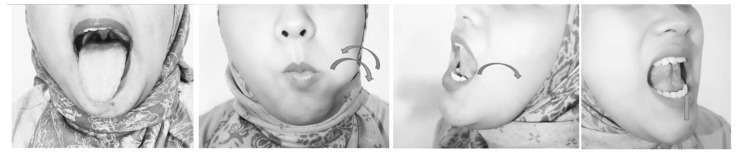
Palatal Exercise.

Continuously pronouncing vocal letters (isometric exercise) trained the palatopharyngeus, palatoglossus, uvula, tensor veli palatine, and levator veli palatine muscles (Fig. 1). Saying vocal letters discontinuously (isotonic exercise) in addition to the muscles mentioned above also trained the lateral wall muscles of the pharynx, such as pharyngeal constrictor muscles. This movement could improve collapse of retropalatal area 10.

### Tongue

**Figure 2:**
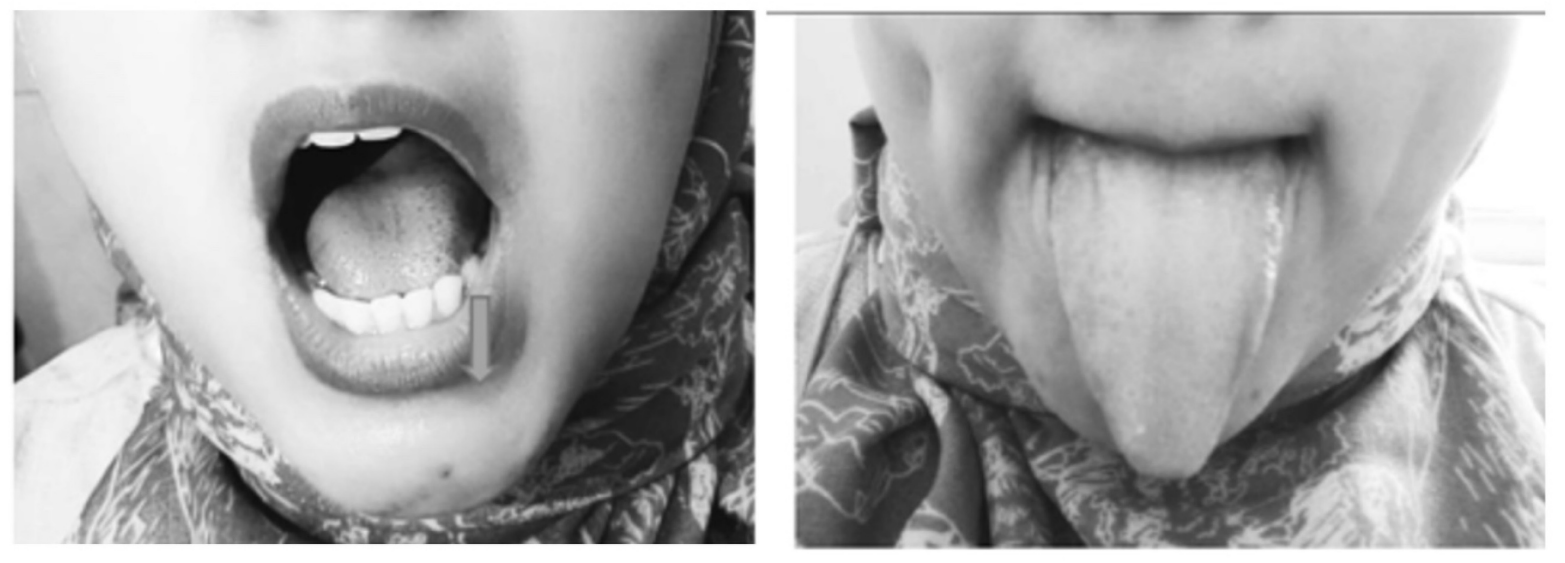
Tongue Exercise.

The movements of sliding the tongue to the upper and side surfaces of the teeth with the tongue towards the left and right (isotonic exercise), placing the tip of the tongue in front of the palate, then sliding the tongue back (isotonic exercise), pressing and sucking the tongue up to the palate (isometric exercise), pressing the back of the tongue while keeping the tip of the tongue touching the front lower teeth (isometric exercise), and sticking out the tongue (isotonic exercise) were movements that trained the genioglossus muscle, to improve collapse of the retroglossal area (Fig. 2).

### Face

**Figure 3:**
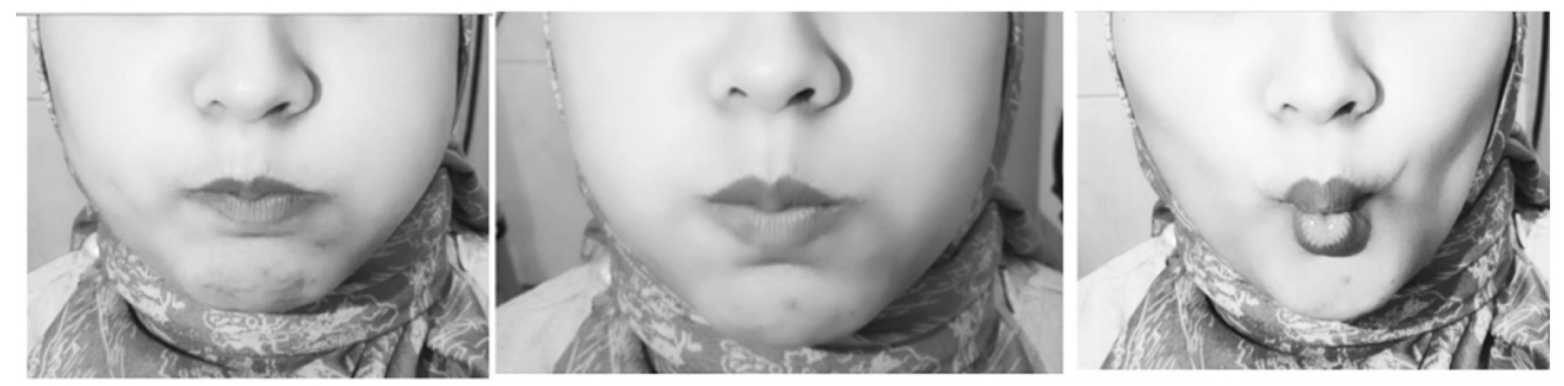
Face Exercise.

Inflating the cheeks with the mouth closed (isometric exercise), gargling (isotonic exercise) to exercise the orbicularis oris muscle, and sucking the cheeks (isometric exercise) to exercise the buccinator muscle. These movements also exercise the zygomaticus major, zygomaticus minor, levator labii superioris, levator anguli oris, lateral pterygoid, and medial pterygoid muscles (Fig. 3).

### Icing

**Figure 4:**
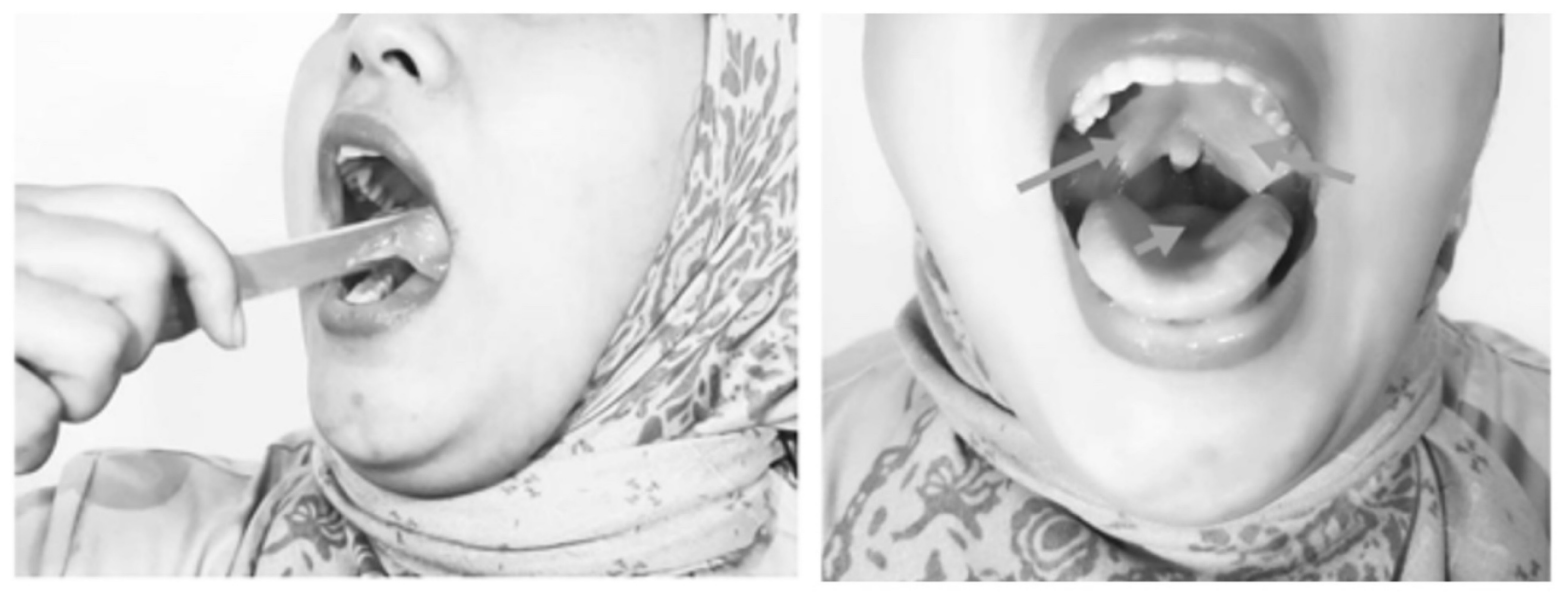
Icing Methods.

Placing ice in the mouth, on the soft palate, palatal arch, base of the tongue, and posterior pharyngeal wall induced a cold, chilling, and numbing sensation, which triggered a reflexive contraction of the palate, tongue, and pharyngeal muscles (Fig. 4).

Patients completed MT exercises by conducting all of this at home, with adherence tracked using exercise log book, submission of clear videos of each exercise, and weekly evaluations by a doctor. Patient compliance was deemed good when exercises were performed more than 75% of the time each week. The study assessed the velopharynx/retropalatal area using FN, daytime sleepiness symptoms via the Epworth Sleepiness Scale (ESS) questionnaire, and snoring intensity and frequency through the Berlin questionnaire after the 6-week training period.

Numerical data analysis before statistical tests was carried out using Kolmogorov-Smirnov normality test (because the subject was < 50) to test whether the data were normally distributed or not normally distributed. Statistical analysis was also conducted according to the study objectives and hypotheses. The significance test to compare the characteristics of the paired study groups with ordinal data was performed using the Wilcoxon test. The significance value used was the p-value, where p ≤ 0.05 was statistically significant, and p > 0.05 was not statistically significant. The data obtained was processed through the Statistical Program for Social Science (SPSS) program version 25.0 for Windows. This study was carried out after approval by the Study Ethics Committee with number LB.02.01/X.6.5/259/2022. All data supporting the results were available within the paper.

## Results

The subjects of this study were originally 18 people but then became 17 people since one patient withdrew. Table 1 explained the characteristics of the study subjects. The average age of the overall respondents was 34.76 ± 7,076 years. For body weight, the average was 85.26 ± 15,671 kg, height had an average of 168.94 ± 5,356, and BMI had an average of 29.97 ± 5,489. Furthermore, the majority were overweight (58.8%), and Table 2 showed that high BMI was not always followed by high ESS scores. Neck circumference had an average of 39.21 ± 1.803 cm with almost the same proportion between groups, and most of the subjects had hypertension (64.75%).

Table 3 showed that blockages occurred not only in retropalatal area but also at various levels of the upper respiratory tract, as many as 100% of people experience retropalatal and retroglossal obstruction. Followed by an obstruction in the nose, namely concha hypertrophy by 70% and deviated septum by 53%, there was a statistically significant widening in retropalatal area before and after MT as shown in Table 4. This was found that all patients who had 25% narrowing had no change after MT to analyze more thoroughly the impact of exercise, but there was a widening of retropalatal area in some subjects who had 50% and 75% retropalatal narrowing (Table 5). Improvement in snoring frequency and intensity significantly (Table 6) mostly were found after MT and there was a decreasing score of ESS after MT significantly as shown in Table 7.

## Discussion

Simple or non-apneic snoring, sometimes referred to as primary snoring, was considered the initial stage of sleep-disordered breathing that did not pose a serious risk to the health of the snorer or other co-sleeper. Despite having a very common phenomenon in the general population, there was no agreement on terminology, hence the authors' understanding of it was restricted 14. In OSA patients, obstruction during sleep occurred mainly at retropalatal level, affecting the anteroposterior and lateral dimensions associated with lateral pharyngeal wall and posterior tongue displacement 15. Kim et al. stated that increased body mass index (BMI) and neck circumference increased the incidence of retropalatal and retroglossal area collapse at the lateral pharyngeal wall level, either the oropharynx or hypopharynx. These results suggested that obesity could lead to an increase in fat in the lateral part of the neck, causing airway collapse 16. Opposite to Kim's reports, this study did not describe that increasing BMI increased the severity of OSA. The data distribution was evenly distributed in each BMI and ESS score group, with mostly overweight patients who had normal ESS scores (Table 4.2). This study also showed subjects had almost the same neck circumference distribution (≥ 40 cm were 47.1%, and < 40 cm were 52.9%) (table 4.1). Furthermore, these results showed that the relationship between ESS score and BMI could vary not only influenced by one factor but depending on many other factors like age, gender, ethnicity, and the presence of other conditions. 17. The variation in BMI and neck circumference factors could affect the results of MT in this study. A previous study showed that while the snore score decreased in the experimental group, MT had no discernible impact on the chronic snoring of obese patients. When therapy was administered without taking into account exclusion criteria that took the severity of sleep breathing difficulties and pharyngeal characteristics, it was unable to treat chronic snoring in obese patients 17.

In this study, 64.7% of subjects had hypertension (Table 4.1), and this finding was in line with the outcome of the Wisconsin Sleep Cohort study, which showed the occurrence of OSA in hypertensive patients (30-50%). However, epidemiologically, OSA and hypertension had a 2-way relationship. These patients also had a high prevalence of hypertension and a higher risk of OSA. Approximately 46% or 53% of OSA patients with a moderate-severe degree (AHI value ≥ 15) became independent risk factors for hypertension, almost 3.2 times higher than individuals without OSA 18,19 This high incidence of hypertension meant that management of snoring patients must be given more attention because hypertension could impact the high morbidity of coronary heart disease and stroke 20.

MT was therapy that could be applied to snoring patients. This exercise involved the tongue, soft palate, lateral wall of the pharynx, and facial muscles, specifically the genioglossus muscle and other pharyngeal dilator muscles. Although the genioglossus muscle was the largest and most potent upper airway dilator, activation alone was not enough to prevent collapse of pharyngeal area 8,21. This exercise aimed to train the muscles involved in the upper airway to prevent collapse by keeping the upper airway muscles open. MT consisted of a combination of oropharyngeal exercises. This was proven to be therapy for OSA patients with mild-moderate degrees affecting increasing pharyngeal muscle tone, which was more physiological, had long-term effects, and had the advantages of remaining non-invasive, cost-effective, and having relatively no side effects 21-24. In addition, Ye et al. reported that those oropharyngeal exercises performed for 6 weeks, 2 times per day for 20 minutes, every day in 196 stroke patients who experienced moderate OSA supervised by speech therapists, showed significant results in improving AHI, snoring index, arousal index, and minimal oxygen saturation. Ye et al. observed that this improvement was related to the addition of retropalatal distance and shortening of the soft palate from magnetic resonance imaging (MRI) results. This showed an improvement in pharyngeal morphology 9. In this study, it was found that all subjects experienced narrowing in the retro-palatal and retro-glossal areas. The MT used as a protocol followed the study of Ye et al 9 and the MT applied consisted of 10 movements, including the soft palate, tongue, and facial muscle movements. This isotonic and isometric exercise could improve sensitivity, proprioception, mobility, and orofacial and pharyngeal coordination. This exercise strengthened and increased oropharyngeal muscle tone, dilating the upper airway during sleep.

This study showed that MT could improve the intensity (100%, p < 0.05) and frequency of snoring (94.1%, p < 0.05). After performing MT in this study, 10 out of 17 people experienced a significant decrease in the percentage of obstruction (as much as 58.8%, p < 0.05), a total of 4 people who did not experience improvement had obstruction > 2 levels, and two people had BMI obesity grade III accompanied by neck circumference ≥ 40 cm. The existence of multi-level obstruction factors on the success of MT needed further evaluation. In addition, it was found that all patients who had 25% narrowing had no change after MT to analyze more thoroughly the impact of exercise, but there was a widening of retropalatal area in some subjects who had 50% and 75% retropalatal narrowing (table 4.6). This was due to the presence of multilevel obstruction (table 4.4) as well as central factors caused by obesity in patients (table 4.1), or other factors that had not been ruled out in this study.

Camacho et al., in a meta-analysis study, showed that MT could reduce AHI by 50% and improve snoring symptoms. Leto et al. 23 showed that oropharyngeal exercises for 3 months in mild-moderate OSA significantly reduced snoring frequency by 36% and snoring intensity by 59%^.^ Maimon et al. 10, in their study, stated that snoring intensity was more severe in patients with BMI > 30 and neck circumference > 40 cm. Furthermore, there was a positive relationship between snoring intensity and AHI value. In patients with AHI < 5 (mild OSA), the average snoring intensity is 52 decibels (moderate intensity) which increased according to the increase in OSA severity. This was an average of 60 decibels at AHI > 50 (very severe OSA) 26. According to a review of the study, MT likely lessened daytime drowsiness and could improve sleep quality within a short term.

This study revealed an improvement in daytime sleepiness symptoms, shown by a significant decrease in the ESS score after 6 weeks of MT, and supported other results. Daytime sleepiness was the most common symptom complained of as drowsiness, which could be accompanied by fatigue or a loss of energy. Sleep breathing disease was caused by 2 main physiological mechanisms, namely intermittent hypoxemia and sleep fragmentation. Intermittent hypoxemia (low blood oxygen levels) caused oxidative neuronal injury to noradrenergic and dopaminergic neurons in the locus coeruleus (LC) and ventral periaqueductal gray (VPG), causing neuronal damage in wake-promoting areas of the brain. Sleep fragmentation could cause damage or degeneration of noradrenergic and orexinergic nerves in the LC, which also caused nerve damage in wake-promoting brain areas. Sleep fragmentation occurred because the patient woke up from sleep repeatedly or briefly, which disrupted normal sleep patterns and prevented the patient from achieving restorative sleep to get restful sleep. After all, this did not reach slow-wave sleep or rapid eye movement phase sleep, which was very important for patients to feel fit and alert during the day. Both of these were associated with daytime sleepiness and fatigue. In addition, fluctuations in oxygen levels due to intermittent hypoxia followed by re-oxygenation also triggered physiological responses, including activation of the sympathetic nervous system and the release of stress hormones. These responses disrupted sleep and led to poor-quality sleep 19, 27,28.

This study had some limitations such as a small sample size and it had no control group for comparison, which could limit generalizability and the ability to make causal inferences about the intervention's effectiveness. The FN examination only used MM technique which was recommended as a dynamic diagnostic method, providing information on the location of upper airway collapse or obstruction starting from the nose, nasopharynx, oropharynx, larynx, and hypopharynx, but it only had a predictive value for OSA severity. Collapse of the lateral pharyngeal level was associated with high AHI values, and the presence of airway collapse on FN examination with MM, specifically lateral pharyngeal wall collapse, required further examination with PSG to confirm improvement in AHI as a sign of snoring severity.

## Conclusion

In conclusion, following the results of this study, the frequency of snoring before performing MT was almost daily, and the intensity of snoring was louder than normal speech. After performing MT, the frequency of snoring was 1-2 times per week, and the intensity was decreased to slightly louder than breathing. All patients reported experiencing complaints of excessive daytime sleepiness concerning their snoring. However, it was determined that these complaints were within normal limits. The oropharynx anatomy referred to as obstruction showed the highest impact by the MT, specifically in retropalatal regions. The application of MT as an intervention has shown the ability to reduce the degree of narrowing of pharyngeal area, the frequency and intensity of snoring, and daytime sleepiness in patients suffering from snoring. Further study must be done using a bigger sample size, with a control group and a long duration of exercise with follow-up to ensure the effect of exercise on the patient.

## Figures and Tables

**Table 1:** Characteristics of the subjects

Variable	n=17
**Age**	
Mean±Std	34.76±7.076
Median	33.00
Range (min-max)	28.00-53.00
**Body Weight (kg)**	
Mean±Std	85.26±15.671
Median	79.00
Range (min-max)	65.00-128.00
**Body Height (cm)**	
Mean±Std	168.94±5.356
Median	170.00
Range (min-max)	161.00-178.00
**BMI (kg/m^2^)**	
Mean±Std	29.97±5.489
Median	28.00
Range (min-max)	24.50-44.20
Normal	1 (5.9%)
Overweight	10 (58.8%)
Obese I	4 (23.5%)
Obese III	2 (11.8%)
**Neck Circumference (cm)**	
Mean±Std	39.21±1.803
Median	39.00
Range (min-max)	35.00-42.00
≥ 40 cm	8 (47.1%)
< 40 cm	9 (52.9%)
**Blood Pressure (mmHg)**	
Hypertension	11 (64.7%)
Normal	6 (35.3%)

**Table 2:** ESS score based on BMI category

BMI (kg/m^2^)	ESS Score: Normal	ESS Score: Mild	ESS Score: moderate
Normal	0% (5,56%)	0%	1 (5,56%)
Overweight	8 (44,4%)	1 (5,56%)	1 (5,56%)
Obese I	3 (16,7%)	0%	1 (5,56%)
Obese II	0%	0%	1 (5,56%)
Obese III	1 (5,56%)	1 (5,56%)	0%

**Table 3:** Multilevel Blockage on Flexible Optic Laryngoscope Examination

Level	Obstructions	Total (p)	Percentage (%)
Nose	Concha Hypertrophy	12	70
	Septal deviation	9	53
Oropharynx	Tonsil hypertrophy	1	5.8
	Retropalatal	17	100
	Retroglossal	17	100
Nasopharynx	Adenoid hypertrophy	6	35
Hypopharynx	Tonsil lingualis	17	100

**Table 4:** Comparison of Retropalatal area Narrowing Before and After Myofunctional Therapy

Variable	Groups		p value
	Before	After	
	n=17	n=17	
Retropalatal area narrowing (%)			0.002*
25	2 (11.8%)	8 (47.1%)	
50	11 (64.7%)	9 (52.9%)	
75	4 (23.5%)	0 (0.0%)	

* p value < 0.05: statistically significant, Wilcoxon test

**Table 5:** Distribution of Percentage Reduction of Retropalatal Narrowing Before and After Myofunctional Therapy

Percentage of retropalatal narrowing before training (%)	Percentage of retropalatal narrowing after training (%)	Total
25	25	2
	50	0
	75	0
50	25	6
	50	5
	75	0
75	25	0
	50	4
	75	0

**Table 6:** Comparison of Snoring Frequency and Intensity Scores Before and After Myofunctional Therapy

Variable	Groups		p value
	Before	After	
	n=17	n=17	
**Frequency of snoring**			0.0001*
Almost every day	12 (70.6%)	0 (0.0%)	
3-4 times/week	5 (29.4%)	4 (23.5%)	
1-2 times/week	0 (0.0%)	10 (58.8%)	
1-2 times/month	0 (0.0%)	3 (17.6%)	
**Intensity of Snoring**			0.0001*
Slightly louder than breathing	0 (0.0%)	13 (76.5%)	
As loud as talking	2 (11.8%)	4 (23.5%)	
Louder than talking	8 (47.1%)	0 (0.0%)	
Very loud, can be heard in the next room	7 (41.2%)	0 (0.0%)	

* p value < 0.05: statistically significant, Wilcoxon test

**Table 7:** Comparison of Epworth Sleepiness Scale (ESS) Questionnaire Before and After Myofunctional Therapy

Variable	Groups		Differences	p value
	Before	After		
	N = 17	N = 17		
Questionnaire ESS Score Value				0.0001*
Mean±Std	9.53±4.474	5.94±3.648	3.59±2.740	
Median	9.00	5.00	4.00	
Range (min-max)	3.00-18.00	0.00-14.00	0.00-9.00	

* p value < 0.05: statistically significant, Wilcoxon test

## Abbreviations

OSA: Obstructive Sleep Apnea
SBQ: STOP-Bang Questionnaire
FN: Flexible Nasolaryngoscopy
MT: Myofunctional Therapy
ENT: Ear, Nose, and Throat
MM: Muller Maneuver
ESS: Epworth Sleepiness Scale
MRI: Magnetic Resonance Imaging
SPSS: Statistical Program for Social Science
ICSD-3: International Classification of Sleep Diseases, Third Edition
LPW: Lateral pharyngeal wall
RC: Retropalatal circumferential
AHI: Apnea-Hypopnea Index
BMI: Body Mass Index
LC: Locus Coeruleus
VPG: Ventral Periaqueductal Gray
